# Corneal graft rejection following COVID-19 vaccine

**DOI:** 10.1038/s41433-021-01671-2

**Published:** 2021-08-23

**Authors:** Konstantinos I. Rallis, Darren S. J. Ting, Dalia G. Said, Harminder S. Dua

**Affiliations:** 1 Department of Ophthalmology, Queen’s Medical Centre, Nottingham, UK; 2 Academic Ophthalmology, Division of Clinical Neuroscience, School of Medicine, University of Nottingham, Nottingham, UK; 3 Research Institute of Ophthalmology, Cairo, Egypt

**Keywords:** Corneal diseases, Immunological disorders, Transplant immunology, Education

## Introduction

COVID-19 has affected >100 million with >2 million deaths worldwide. A number of effective mRNA-based [1] and viral vector-based vaccines [2] have been developed and deployed. However, as COVID-19 vaccines have been shown to induce strong immune responses [3], there remains a hypothetical concern whether the vaccine could increase the risk of transplant rejection in non-immunosuppressed patients. In this report, we highlight a case of acute corneal allograft rejection shortly after the administration of COVID-19 vaccine.

## Case description

In February 2021, a 68-year-old woman attended the eye casualty with a 1-day history of left painful red eye and rapid deterioration of vision. Four days earlier, she received her first dose of mRNA-based BNT162b2 COVID-19 vaccine (Pfizer-BioNtech). On the next day, she developed moderate systemic reactions, including chills, myalgia, and tiredness, followed by unexpected ocular symptoms on the third day.

Her past ocular history included a bilateral lamellar Descemet Stripping Automated Endothelial Keratoplasty (DSAEK) for Fuchs’ corneal endothelial dystrophy and a left re-do penetrating keratoplasty (PKP) for failed DSAEK in October 2020. There was no other relevant past ocular/medical history. At 2-month post-PKP, her left eye best-corrected visual acuity (BCVA) was 6/18 with a clear corneal graft. She was maintained on topical prednisolone 0.5% QID for the left eye and dexamethasone 0.1% OD for the right eye.

**Fig. 1:**
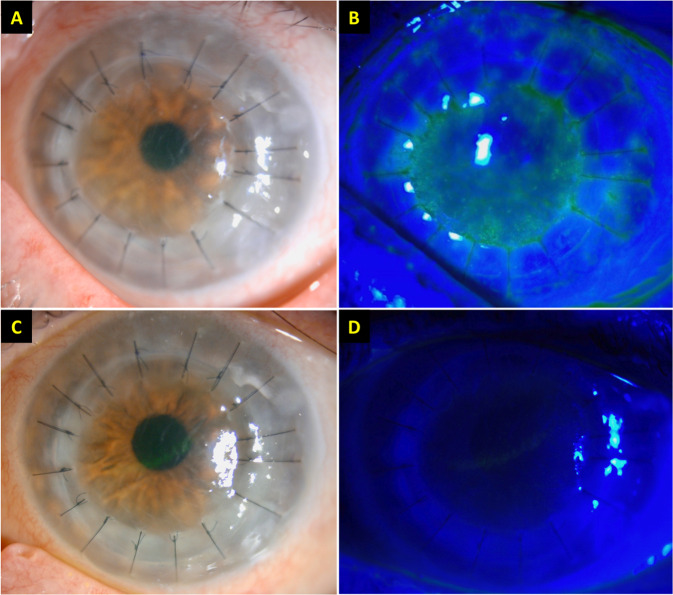
A case of acute corneal endothelial graft rejection after COVID-19 vaccine. **A, B** Slit-lamp photography demonstrating conjunctival hyperaemia, corneal graft haze, diffuse corneal epithelial, and stromal oedema (within the graft), Descemet’s folds, scattered keratic precipitates (KPs), and 1+ cells in anterior chamber. An unusual distribution of fluorescein staining with coarse punctate epitheliopathy over the corneal graft was observed. The central corneal thickness (CCT) was 730 μm. **C, D** At 3-week post treatment, the corneal graft rejection was successfully treated with considerable improvement in the graft transparency, reduction in epithelial and stromal oedema, and resolution of epitheliopathy and anterior chamber inflammation. The best-corrected visual acuity improved to 6/12, with a CCT of 609 μm.

At presentation, her left BCVA was counting fingers. Slit-lamp examination confirmed the diagnosis of acute corneal endothelial graft rejection, evidenced by conjunctival hyperaemia, diffuse corneal punctate staining, corneal graft oedema, Descemet’s folds, scattered keratic precipitates, and anterior chamber activity (Fig. 1). The right corneal graft remained healthy.

The patient was immediately treated with hourly topical dexamethasone 0.1% and a week of oral acyclovir 400 mg 5x/day (to cover for any possible underlying herpes simplex keratitis), with no treatment modification in the right eye. Significant improvement was noted by 3-week post-treatment with complete resolution of corneal graft rejection (Fig. 1C, D).

## Discussion

Corneal graft rejection after vaccination is rare, with most evidence so far being linked to influenza vaccination. To our best knowledge, this is the second report of corneal allograft rejection following COVID-19 vaccine [4].

COVID-19 vaccines have been shown to induce SARS-CoV-2 neutralising antibodies and elicit strong Th1-biased CD4+ responses in human [3]. CD4+ Th1 cells have been shown to be the main mediators of corneal graft rejection [5], which could have contributed to the graft rejection. Intriguingly, corneal graft rejection only occurred in the left eye despite the presence of bilateral grafts. This may be attributed to the more recent history of left eye surgery and a repeat graft, which is known to have a higher risk of rejection. Although a direct cause-and-effect relationship remains to be established, it would be prudent to defer elective corneal transplant by 3–6 months after the second dose of COVID-19 vaccines and to consider institution of intensive topical steroids (6–8 times a day) in grafted eyes at the time of the first dose of vaccine until at least a month after the second dose, as the immune response peaks on 7–28 days post vaccine [3].
